# Tannic Acid-Steeped Corn Grain Modulates *in vitro* Ruminal Fermentation Pattern and Microbial Metabolic Pathways

**DOI:** 10.3389/fvets.2021.698108

**Published:** 2021-10-28

**Authors:** F. F. Zhao, X. Z. Zhang, Y. Zhang, Mawda Elmhadi, Y. Y. Qin, H. Sun, H. Zhang, M. Z. Wang, H. R. Wang

**Affiliations:** ^1^Laboratory of Metabolic Manipulation of Herbivorous Animal Nutrition, College of Animal Science and Technology, Yangzhou University, Yangzhou, China; ^2^Joint International Research Laboratory of Agriculture and Agri-Product Safety, The Ministry of Education of China, Yangzhou University, Yangzhou, China; ^3^College of Animal Science and Veterinary Medicine, Heilongjiang Bayi Agricultural University, Daqing, China; ^4^Jiangsu Coastal Area, Institute of Agricultural Sciences, Yancheng, China

**Keywords:** tannic acid treated corn, rumen fermentation characteristics, ruminal microbiota, high-throughput sequencing, rumen acidosis

## Abstract

This study investigated the effects of tannic acid (TA)-treated corn on changes in ruminal fermentation characteristics and the composition of the ruminal bacterial community *in vitro*. Ruminal fluid was obtained from three rumen-fistulated goats fed a 60:40 (forage/concentrate) diet. The batch cultures consisted of 25 ml of strained rumen fluid in 25 ml of an anaerobic buffer containing 0.56 g of ground corn, 0.24 g of soybean meal, 0.10 g of alfalfa, and 0.10 g of oat grass. Ground corn (2 mm) was steeped in an equal quantity (i.e., in a ratio of 1:1, *w*/*v*) of water alone (Con), 15 (TA15), 25 (TA25), and 35 g/l (TA35) TA solution for 12 h. After incubation for 24 h, TA-treated corn linearly increased (*P* <0.05) ruminal pH and the molar proportion of acetate, but linearly reduced (*P* <0.05) total volatile fatty acids and the molar proportion of butyrate compared with the Con treatment. Illumina MiSeq sequencing was used to investigate the profile changes of the ruminal microbes. A principal coordinates analysis plot based on weighted UniFrac values revealed that the structure of the ruminal bacterial communities in the control group was different from that of the TA-treated corn groups. The results of changes in the rumen bacterial communities showed that TA-treated corn linearly enriched (*P* <0.05) *Rikenellaceae_RC9_gut_group*, but linearly reduced (*P* <0.05) *Ruminococcaceae_NK4A214_group, Ruminococcus_2*, and *unclassified_o__Clostridiales*. Functional prediction of ruminal microbiota revealed that the TA-treated corn linearly decreased ruminal microbiota function of utilizing starch through pyruvate metabolism. In conclusion, TA-treated corn can modulate the rumen fermentation characteristics, microbial composition, and metabolic pathways, which may be potentially useful for preventing the occurrence of ruminal acidosis.

## Introduction

In the current intensive rearing system, ruminants are often fed a high-grain diet or easily fermentable carbohydrates to maximize energy intake and support fattening and milk production. Excessive amounts of carbohydrates cause volatile fatty acid (VFA) accumulation, ruminal pH depression, and ruminal microbiota dysbiosis, which could increase the risk of developing subacute rumen acidosis (SARA) accompanied with laminitis, mastitis, liver abscesses, and digestive tract damage (1, 2). Due to the negative effect of high-grain diets, which decreased the long-term production of ruminants, several dietary strategies such as sodium bicarbonate buffer (3), dicarboxylic acids (4), probiotics (5), and thiamine (6) have been used to prevent the occurrence of SARA. In addition, substantial research efforts have been made over the years to develop grain processing technologies to modulate the rumen degradability of grain, aiming to improve grain feed efficiency and its health benefits (7–9).

Recently, organic acids saved as a modifier have been proposed for use in chemical processing of easily fermentable cereal grains to change ruminal starch degradation site and patterns and then alleviate the negative effects of SARA [as reviewed by (10)]. More specially, studies by Iqbal et al. (11), Iqbal et al. (12), and Iqbal et al. (13) showed that feeding barley grain steeped in 0.5% lactic acid increased starch resistance to microbial fermentation, lowered ruminal concentration of VFA, lactic acid, and lipopolysaccharide (LPS) in dairy cows. Treatment of barley or corn grain with other organic acids, such as citric acid, also increases ruminal-resistant starch (14–17). As for tannic acid (TA), Deshpande et al. (18) reported that at room temperature, the binding of TA to starches (legume starches and potato starch) and starch fractions (amylose and amylopectin) lowered the *in vitro* digestibility by 8.8–17.0% with porcine pancreatic α-amylase. The protective effect of TA against ruminal degradation in cereal grains has been scarcely explored. A study by Martínez-Moya et al. (8) using TA to protect barley meal against ruminal degradation showed limited microbial hydrolysis of endosperm cell walls in TA-treated barley. It proved that TA might affect slowing ruminal disappearance of barley, which benefits to improve starch utilization by ruminants. Corn is a starch-rich grain that is widely used as animal feed in China. A high intake of corn-based diet increases the starch digestibility in the rumen. Thus, feeding high-corn diet can induce SARA in dairy cows and goats (19–21). Accordingly, we chose TA as a modifier of the fermentation characteristics of corn. We hypothesized that ground corn steeped in TA solution would reduce the rate of starch ruminal digestion under high-corn substrates, which will improve the ruminal pH and reduce the risk of SARA. Most studies to date have focused on the effects of organic acid-treated cereal grains on the rumen fermentation pattern; however, limited information was reported from changes in ruminal microbial point of view. Thus, the present study aimed to investigate the effects of TA-treated corn with different concentrations on the fermentation characteristics, the ruminal microbial composition, the correlations between microbial communities and ruminal variables as well as the effects of TA-treated corn on the ruminal microbial metabolic pathways.

## Materials and Methods

All animal experiments were conducted in line with the Animal Protection Law based on the Guidelines for the Care and Use of Laboratory Animals approved by the Ethics Committee of Yangzhou University (SXXY2015-0054).

### Corn Sample Processing

Ground corn grain was passed through a 2-mm mesh sieve and then treated with four different tannic acid concentrations (purity 98%, Macklin Biochemical Technology Co., Ltd, Shanghai, China): 0, 15, 25, and 35 g tannic acid/kg corn. In the TA-treated corn, 100 ml of an aqueous solution of tannic acid with concentrations of 1.5, 2.5, and 3.5 g tannic acid/100 ml were thoroughly mixed with 100 g of ground corn, respectively. In the control corn, 100 g of ground corn was soaked in an equal volume (100 ml) of distilled water. After 10 min of stirring, all the treatments were soaked for 12 h and then oven dried at 45°C before being mixed with other substrates.

### *In vitro* Batch Fermentations

The effects of TA-steeped corn on rumen microbial fermentation and bacterial communities were evaluated in *in vitro* batch culture of rumen fluid supplied with a 20:80 forage/concentrate diet [17.49 CP, 21.01% NDF, 12.44% ADF, and 34.94% starch, dry matter (DM) basis] to simulate *in vivo* SARA induced by high concentrate diet. The substrate (DM basis) consisted of 560 mg ground corn, 240 mg soybean, 100 mg alfalfa, and 100 mg oat grass. Corn in the control group and TA groups were steeped in water and tannic acid solution for 12 h, respectively. Ruminal fluid was obtained from three rumen-fistulated goats (30.25 ± 1.25 kg of body weight) fed a 60:40 forage/concentrate diet (Table 1). The animals were housed in individual pens with free access to water and fed twice daily at 0800 h and 1,800 h. Rumen fluid was obtained immediately before morning feeding and strained through four layers of cheesecloth, mixed in a 1-to-1 proportion with phosphate-bicarbonate buffer (22), purged with anaerobic-grade CO_2_ (<2 ppm O_2_), and standardized at pH 7.0 with 3 N HCl (23). Immediately after inoculation, the bottles were gassed with CO_2_ before sealing with rubber stoppers. Incubations were conducted at 39°C in a shaking water bath. There were nine replicate bottles in both the TA treatment groups and the control group. The fermentation process was conducted in 100-ml sterile glass bottles containing 50 ml of culture fluid with 1 g of the diet ground through a 2-mm screen. After 24 h, the culture fluid of each bottle was filtered through four layers of cheesecloth and the pH was determined immediately. Filtered liquor samples for VFAs, lactate acid, and microbial DNA extraction were collected for analyses.

**Table 1:** Ingredients and nutritional composition of basal experimental diet offered to rumen fluid donor goats.

**Item**	**Composition of experimental diet**
**Ingredient (% DM)**	
Oat grass hay	40.00
Alfalfa hay	20.00
Grounded corn	34.30
Soybean meal	3.80
Limestone	0.40
Salt	0.50
Premix^a^	1.00
**Nutrient composition**	
Metabolizable energy (MJ/kg DM)^b^	8.36
Crude protein (% DM)^c^	10.79
Neutral detergent fiber (% DM)^c^	41.54
Starch (% DM)^c^	21.02
Calcium (% DM)^c^	0.57
Phosphorus (% DM)^c^	0.26

a *Contained 7 g/kg of Fe, 8 g/kg of Zn, 5 g/kg of Mn, 1.2 g/kg of Cu, 130 mg/kg of I, 27 mg/kg of Se, 45 mg/kg of Co, 1,600,000 IU/kg of vitamin A, 150,000 IU/kg of vitamin D, and 62,000 IU of vitamin E*.

b *Values was calculated*.

c *Values based on analysis*.

### Chemical Analysis

The chemical analysis of the diet for rumen fluid donor goats was analyzed for DM (ISO 6496) and CP (ISO 15670), according to the Association of Analytical Chemists (24). Neutral detergent fiber contents were determined according to the procedure of Van Soest (25). Starch content was determined according to the procedure of Hall (26). Calcium and phosphorus were measured by inductively coupled plasma emission spectroscopy using an Atom Scan 25 Plasma Spectroscopy (Thermo Jarrell Ash Corp., Grand Junction, CO) after acid digestion.

### Rumen Fluid Sampling and Analysis

The ruminal pH values in each bottle was determined at the end of the 24-h fermentation with a pH meter (FE20, METTLER TOLEDO, USA). The VFA concentrations were analyzed according to the gas chromatography (GC-14B, Shimadzu, Japan) method proposed by Qin et al. (27) with slight modifications. Briefly, 1 ml ruminal fluid supernatant was added to 0.2 ml metaphosphoric acid solution (25%) and vortexed for 30 s. Then, samples were centrifuged at 12,000 × g for 15 min at 4°C. Samples were filtered by a 0.45-μm filter and then transferred into a capillary column GC (30 m ×1.0 μm ×0.53 mm, Agilent, Netherlands; column temperature, 150°C; injector temperature, 200°C; and detector temperature, 200°C). The concentration of lactate acid was analyzed following the method described by Barker and Summerson (28).

### DNA Extraction and Illumina MiSeq Sequencing, Sequence Processing, and Analysis

Microbial DNA was extracted using the FastDNA® soil DNA Kit (Omega Bio-tek, Norcross, GA, USA) according to manufacturer's protocol. The final DNA concentration and purification were determined by NanoDrop 2000 UV-vis spectrophotometer (Thermo Scientific, Wilmington, USA), and DNA quality was checked by 1% agarose gel electrophoresis. The V3-V4 hypervariable regions of the bacterial 16S rRNA gene were amplified with primers 338F (5′-ACTCCTACGGGAGGCAGCAG-3′) and 806R (5′-GGACTACHVGGGTWTCTAAT-3′) by a thermocycler PCR system (GeneAmp 9700, ABI, USA). The PCR reactions were conducted using the following program: 3 min of denaturation at 95°C, 27 cycles of 30 s at 95°C, 30 s for annealing at 55°C, 45 s for elongation at 72°C, and a final extension at 72°C for 10 min. Polymerase chain reactions were performed in triplicate 20-μl mixture containing 4 μl of 5x FastPfu Buffer, 2 μl of 2.5 mM dNTPs, 0.8 μl of each primer (5 μM), 0.4 μl of FastPfu Polymerase, and 10 ng of template DNA. The resulted PCR products were extracted from a 2% agarose gel and further purified using the AxyPrep DNA Gel Extraction Kit (Axygen Biosciences, Union City, CA, USA) and quantified using QuantiFluor TM-ST (Promega, USA) according to the manufacturer's protocol. Amplicon libraries were generated using NEXTFLEX® Rapid DNA-Seq Kit (Bioo Scientific, USA) following the manufacturer's recommendations. The paired-end sequence (2 ×300 bp) was conducted on an Illumina MiSeq platform (Illumina, San Diego, USA).

Raw fastq files were quality-filtered by Trimmomatic and merged by FLASH with the following criteria: (i) the reads were truncated at any site receiving an average quality score <20 over a 50-bp sliding window. (ii) Sequences whose overlap being longer than 10 bp were merged according to their overlap with mismatch no more than 2 bp. (iii) Sequences of each sample were separated according to barcodes (exactly matching) and primers (allowing two-nucleotide mismatching), and reads containing ambiguous bases were removed. Operational taxonomic units (OTUs) were clustered with 97% similarity cutoff using UPARSE (version 7.1) (29) with a novel greedy algorithm that performs chimera filtering and OTU clustering simultaneously. The taxonomy of each 16S rRNA gene sequence was analyzed by RDP Classifier against the Silva (SSU138) 16S rRNA database using a confidence threshold of 70% (30). OTUs were used to generate rarefaction curves and Shannon–Wiener curves. The bacterial community diversity by calculating the diversity estimator indices of Shannon–Wiener, Simpson, Chao1, and abundance-based coverage estimator (ACE) (31) using Mothur version v.1.30.2 (32). The weighted UniFrac distance method was used to perform a principal coordinate analysis (PCoA), and an analysis of similarity (ANOSIM) in QIIME with 999 permutations (33) was conducted to assess significant differences between samples.

### Functional Prediction of Ruminal Microbiome

Functional capacity of the ruminal microbial community was predicted using the PICRUSt (phylogenetic investigation of communities by reconstruction of unobserved states) software package (http://picrust.github.io/picrust) (34). The significant difference in KEGG (Kyoto Encyclopedia of Genes and Genomes) ortholog (KO) abundances among treatments was identified and analyzed using the KEGG Mapper pathway search function.

### Statistical Analyses

Data of ruminal fermentation profile, the rumen bacterial communities and their functional changes, were analyzed using one-way analysis of variance (ANOVA) procedure of SAS (SAS Inst. Inc., Cary, NC). The differences among treatment means were determined using Duncan's multiple range tests. Correlations between bacterial communities and ruminal fermentation parameters as well as correlations between bacterial communities and altered metabolic pathways were assessed by Pearson's correlation test. *P*-values below 0.05 were regarded as statistically significant.

## Results

### *In vitro* Ruminal Fermentation Parameters

The ruminal pH value, VFA concentrations, and individual VFA profiles are presented in Table 2. TA-treated corn linearly increased (*P* <0.05) in ruminal pH and linearly decreased (*P* <0.05) the total VFA concentration. With the respect to individual VFA, TA-treated corn linearly increased (*P* <0.05) the molar proportion of acetate and the ratio of acetate to propionate, but linearly decreased (*P* <0.05) the molar proportion of butyrate. The molar proportion of propionate was the highest in TA15 treatment, followed by TA35, TA25 and Con treatments. The molar proportion of valerate was linearly decreased (*P* <0.05) in TA treatments. The molar proportion of isovalerate was the highest in TA25 treatment, and there were no significant effect (*P* > 0.05) among TA15, TA35, and Con treatments. In the present study, the result showed that lactate and isobutyrate were not affected (*P* > 0.05) by TA treatments.

**Table 2:** Effects of tannic acid concentration treatments of ground corn on *in vitro* fermentation characteristics and lactate concentration.

**Items**	**TA treatments**				**SEM**	***P*** **-value**	
	**0**	**0.015**	**0.025**	**0.035**		**Linear**	**Quadratic**
pH	5.79	5.90	5.96	5.94	0.012	<0.001	<0.001
TVFA(mmol/l)	163.91	160.65	159.72	159.57	0.640	0.013	0.199
Acetate (%)	56.40	57.58	58.43	58.85	0.192	<0.001	0.101
Propionate (%)	22.34	22.74	22.46	22.70	0.066	0.176	0.521
Butyrate (%)	15.82	14.30	13.55	13.09	0.182	<0.001	<0.001
Isobutyrate (%)	1.29	1.27	1.32	1.26	0.011	0.570	0.340
Valerate (%)	1.50	1.45	1.48	1.43	0.007	0.001	0.589
Isovalerate (%)	2.65	2.67	2.77	2.66	0.011	0.052	0.007
Acetate/propionate	2.52	2.53	2.61	2.60	0.013	0.012	0.724
Lactate (mmol/l)	0.23	0.24	0.24	0.23	0.007	0.743	0.121

*Con, water treatment; TA, tannic acid treatment; SEM, standard error of the mean; TVFA, total volatile fatty acids. Acetate (%), propionate (%), isobutyrate (%), butyrate (%), isovalerate (%), and valerate (%) imply the molar proportion of each to that of the TVFA. Data represent the means of 9 replicates (n = 9)*.

### Diversity, Richness, and Composition of Bacterial Communities in Rumen Fluid

**Figure 1:**
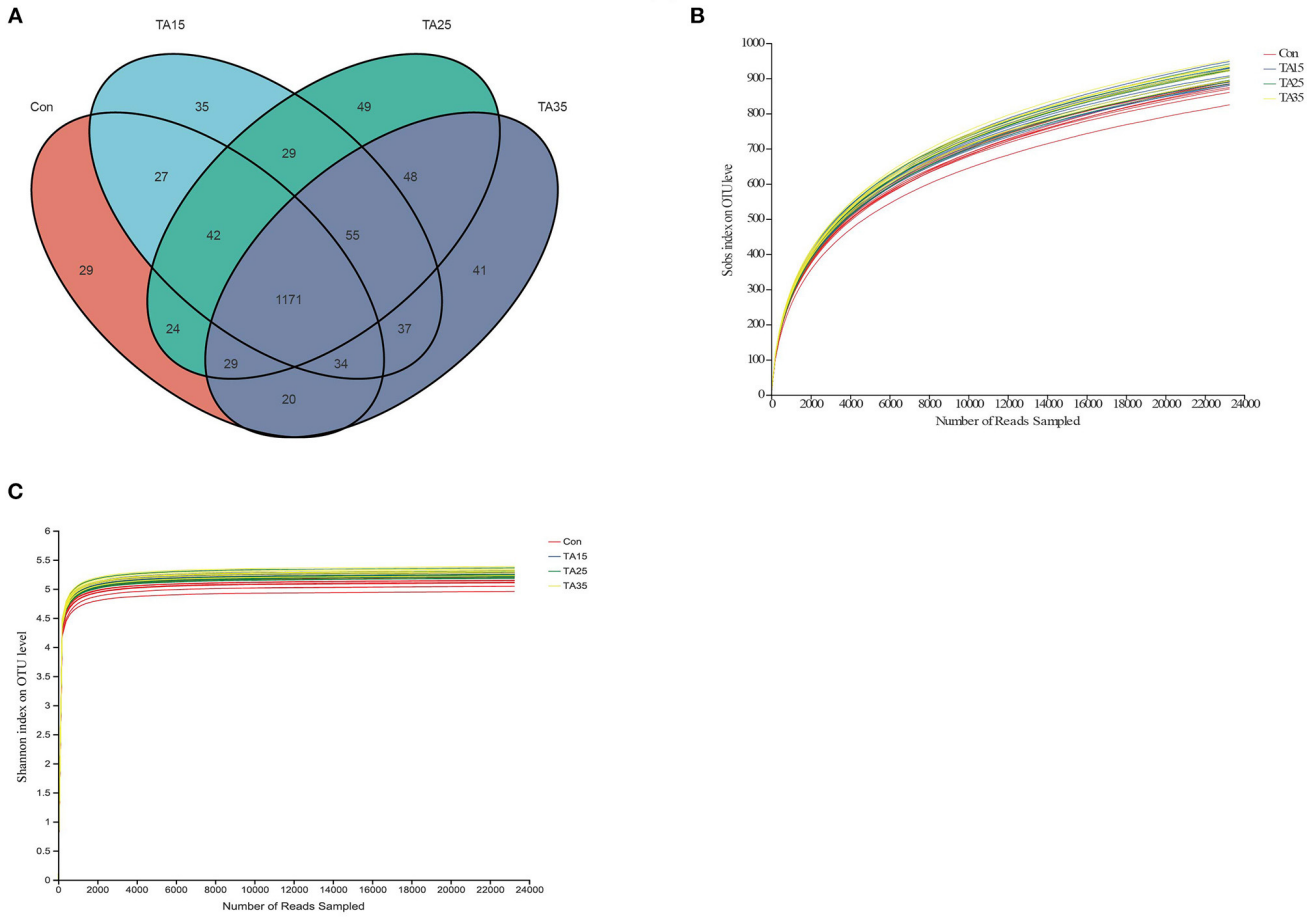
Ruminal microbial operational taxonomic units (OTUs) among different groups. CON, corn steeped in water; TA15, corn steeped in tannic acid solution of 15 g/l; TA25, corn steeped in tannic acid solution of 25 g/l; TA35, corn steeped in tannic acid solution of 35 g/l. **(A)** Venn diagram of ruminal bacterial OTUs. **(B)** Bacterial rarefaction curves and **(C)** Shannon curves based on OTUs used to assess the depth of coverage for each sample.

A total of 2,197,647 reads were obtained for the bacterial 16S rRNA genes by sequencing, with a mean length of 413 bp. Quality filtering at 97% similarity resulted in 837,216 high-quality sequences, which clustered in 1,670 OTUs. A Venn diagram revealed that the numbers of unique OTU for the Con, TA15, TA25, and TA35 treatments were 29, 35, 49, and 41, respectively. Moreover, the Venn profile also revealed that 1,171 OTUs (around 70% of total OTUs) were shared among the four treatments, revealing the presence of a core microbiota (Figure 1A). The rarefaction curve showed that the sequencing work was relatively comprehensive in covering the bacterial diversity, as the rarefaction curves tended to approach saturation (Figure 1B). The Shannon curves indicated that dataset from the diversity analysis was large enough to reflect the bacterial diversity information of samples (Figure 1C).

**Figure 2:**
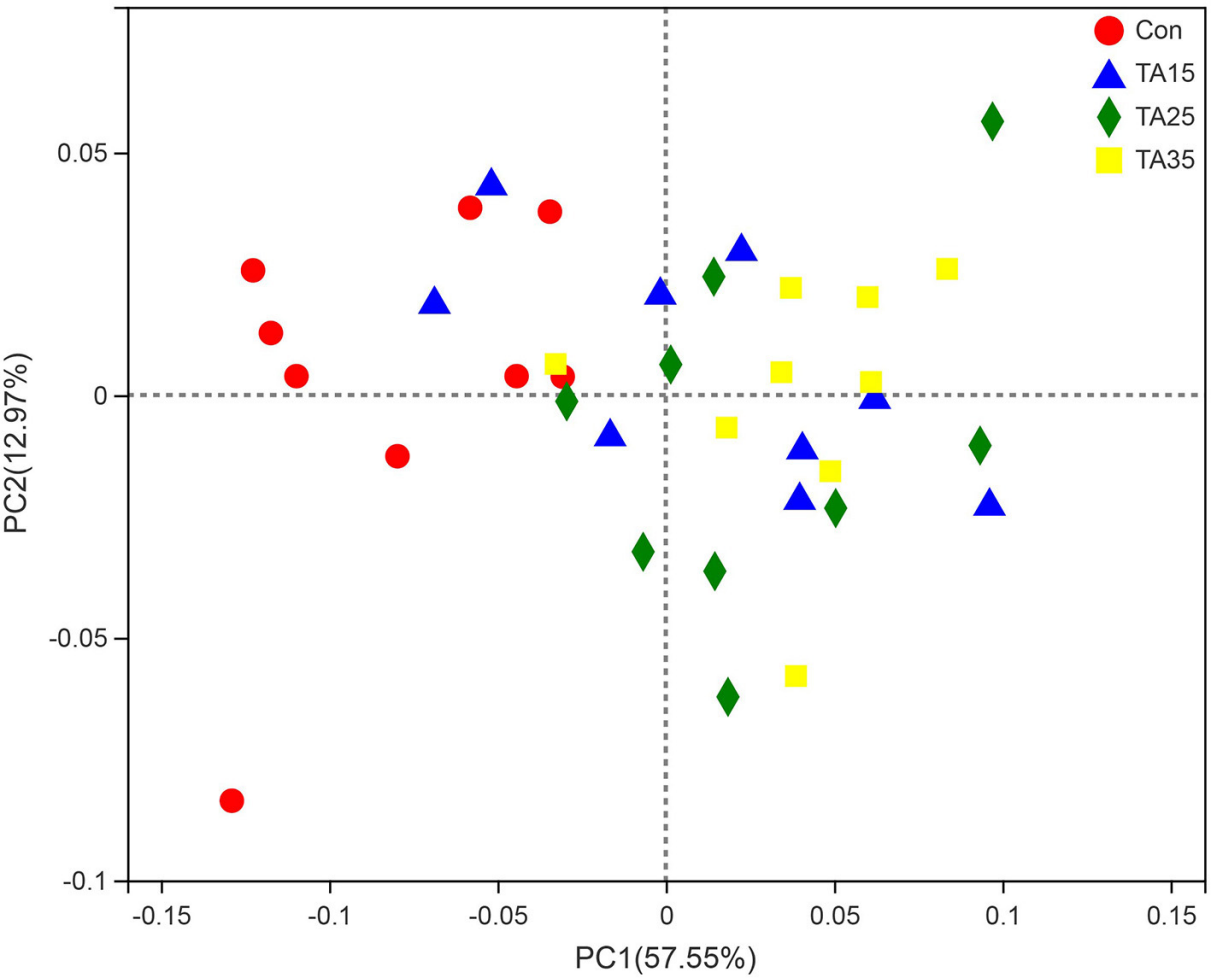
Principal coordinate analysis (PCoA) of bacterial community structures of the ruminal microbiota in CON (red circles), TA15 (blue triangle), TA25 (green diamond), and TA35 (yellow square) groups. PCoA plots were constructed using the weighted UniFrac method.

As shown in Table 3, there were no significant differences (*P* > 0.05) in Good's coverage and ACE index among the groups. TA treatments significantly increased the OTU numbers and Shannon index, but significantly decreased (*P* <0.05) the Simpson index compared with the Con group. The results of PCoA with weighted UniFrac distances indicated that the Con group largely separated from the TA treatments. The PCoA axis 1 accounted for 57.55% of the variation, and the PCoA axis 2 accounted for 12.97% of the variation (Figure 2).

**Table 3:** Effects of tannic acid concentration treatments of ground corn on the number of observed species and alpha diversity of bacterial community structures.

**Items**	**TA treatments**				**SEM**	***P*** **-value**	
	**0**	**0.015**	**0.025**	**0.035**		**Linear**	**Quadratic**
OTU	876	910	917	918	11.2509	0.001	0.050
Good's coverage	0.99	0.99	0.99	0.99	0.0003	0.389	0.885
Shannon index	5.12	5.26	5.25	5.29	0.0277	<0.001	0.030
ACE	1106	1125	1143	1138	19.9173	0.078	0.387
Chao 1	1127	1137	1166	1171	24.6357	0.048	0.900
Simpson index	0.017	0.013	0.014	0.012	0.0008	<0.001	0.038

*Con, water treatment; TA, tannic acid treatment; SEM, standard error of the mean; OUT, operational taxonomic units; ACE, abundance-based coverage estimator. Data represent the means of 9 replicates (n = 9)*.

**Figure 3:**
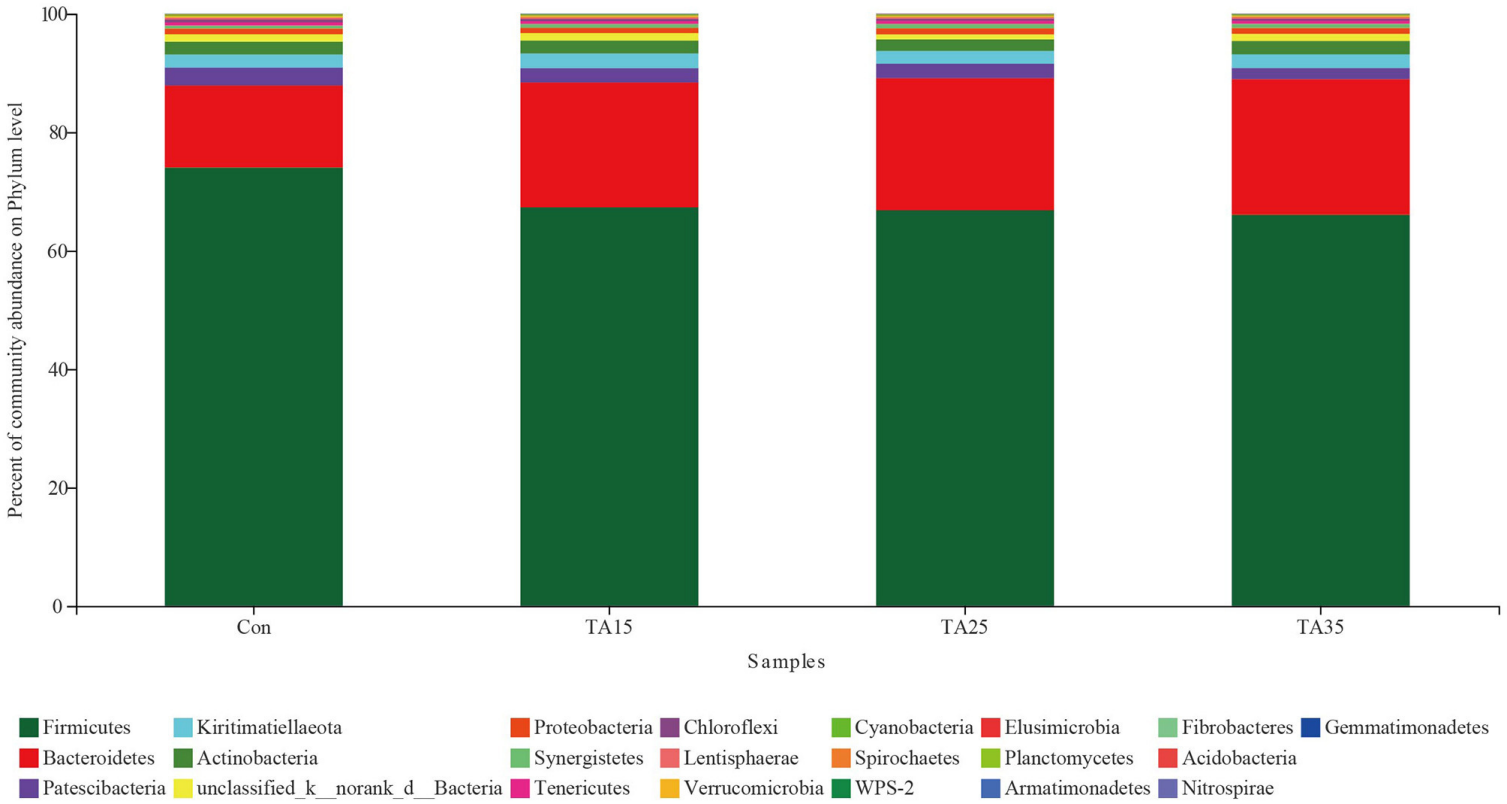
Percentage composition of the total phyla in rumen fluid. CON, corn steeped in water; TA15, corn steeped in tannic acid solution of 15 g/l; TA25, corn steeped in tannic acid solution of 25 g/l; TA35, corn steeped in tannic acid solution of 35 g/l.

Overall, 22 phyla were identified across all samples. Bacteroidetes and Firmicutes were the two dominant phyla (Figure 3). The proportions of *Verrucomicrobia, Tenericutes, Synergistetes, Spirochaetes, Lentisphaerae, Cyanobacteria, Chloroflexi, WPS-2, Planctomycetes*, and *Elusimicrobia* phyla were less than 1% of total microbial community, while *Nitrospirae, Gemmatimonadetes, Fibrobacteres, Armatimonadetes*, and *Acidobacteria* phyla were not consistently present in all of the ruminal samples.

### Changes in the Rumen Bacterial Communities

The 10 most relatively abundant bacterial phyla are shown in Table 4. The relative abundance of *Firmicutes* was the highest in the Con treatment, and the lowest in the TA35 treatment (*P* <0.05), but there was no significant difference (*P* > 0.05) between the TA15, TA25, and TA35 treatments. The relative abundance of *Bacteroidetes* was lower in the Con treatment (*P* <0.05) than the TA15, TA25, and TA35 treatments. The relative abundance of Patescibacteria was the highest in the control treatment, followed by the TA15 and TA25 treatments, and the lowest in the TA35 treatment (*P* <0.05). There were no significant shifts (*P* > 0.05) detected in the relative abundance of *Kiritimatiellaeota, Actinobacteria, unclassified_k__norank_d__Bacteria, Proteobacteria, Synergistetes, Tenericutes*, and *Chloroflexi*.

**Table 4:** Effects of tannic acid concentration treatments of ground corn on the 10 most relatively abundant bacterial phyla.

**Items**	**TA treatments**				**SEM**	***P*** **-value**	
	**0**	**0.015**	**0.025**	**0.035**		**Linear**	**Quadratic**
*Firmicutes*	73.94	67.23	67.54	60.05	4.623	0.008	0.358
*Bacteroidetes*	14.29	21.94	22.90	26.33	3.231	0.003	0.049
*Patescibacteria*	3.05	2.46	2.46	1.92	0.244	0.000	0.875
*Kiritimatiellaeota*	2.25	2.49	2.17	2.32	0.289	0.987	0.737
*Actinobacteria*	2.18	2.15	1.91	2.28	0.230	0.967	0.288
*unclassified_k__norank_d__Bacteria*	1.22	1.23	0.85	1.22	0.264	0.627	0.468
*Proteobacteria*	0.99	0.91	1.00	0.96	0.087	0.960	0.595
*Synergistetes*	0.49	0.64	0.68	0.76	0.135	0.086	0.342
*Tenericutes*	0.55	0.46	0.52	0.53	0.076	0.844	0.309
*Chloroflexi*	0.47	0.37	0.36	0.33	0.087	0.879	0.653

*Con, water treatment; TA, tannic acid treatment; SEM, standard error of the mean. Data represent the means of 9 replicates (n = 9)*.

The 10 most relatively abundant bacterial genera are shown in Table 5. The relative abundance of *Christensenellaceae_R-7_group, Ruminococcaceae_NK4A214_group, Ruminococcus_2*, and *unclassified_ o__ Clostridiales* was higher (*P* <0.05) in the Con treatment than the TA15, TA25, and TA35 treatments. Moreover, the relative abundance of *Rikenellaceae_RC9_gut_group* was lower (*P* <0.05) in the Con treatment than the TA15, TA25, and TA35 treatments. The relative abundance of *Succiniclasticum* and *Quinella* increased with TA treatment, where it reached a significant level between TA25, TA35, and the Con treatment. The relative abundance of *Prevotella_1, norank_f__Muribaculaceae*, and *Family_XIII_AD3011_group* was not affected (*P* > 0.05) by any treatments.

**Table 5:** Effects of tannic acid concentration treatments of ground corn on the 10 most relatively abundant bacterial genera.

**Items**	**TA treatments**				**SEM**	***P*** **-value**	
	**0**	**0.015**	**0.025**	**0.035**		**Linear**	**Quadratic**
*Christensenellaceae_R-7_group*	14.44	11.97	11.98	11.57	0.717	0.000	0.087
*Ruminococcaceae_NK4A214_group*	12.92	10.05	9.14	8.78	0.894	0.000	0.136
*Succiniclasticum*	5.34	7.13	8.48	8.66	1.36	0.013	0.479
*Rikenellaceae_RC9_gut_group*	3.31	6.01	6.95	7.10	0.859	0.000	0.108
*Prevotella_1*	3.77	6.04	5.46	6.09	1.193	0.077	0.368
*Ruminococcus_2*	5.78	4.62	4.68	4.46	0.427	0.004	0.179
*Quinella*	2.71	4.33	5.54	5.38	0.976	0.004	0.356
*norank_f__Muribaculaceae*	3.13	3.82	4.19	4.04	0.518	0.057	0.358
*unclassified_o_Clostridiales*	4.65	3.45	3.13	3.12	0.318	0.000	0.036
*Family_XIII_AD3011_group*	3.59	3.13	2.87	3.32	0.329	0.237	0.085

*Con, water treatment; TA, tannic acid treatment; SEM, standard error of the mean. Data represent the means of 9 replicates (n = 9)*.

### Correlations Between Bacterial Communities and Ruminal Variables

**Figure 4:**
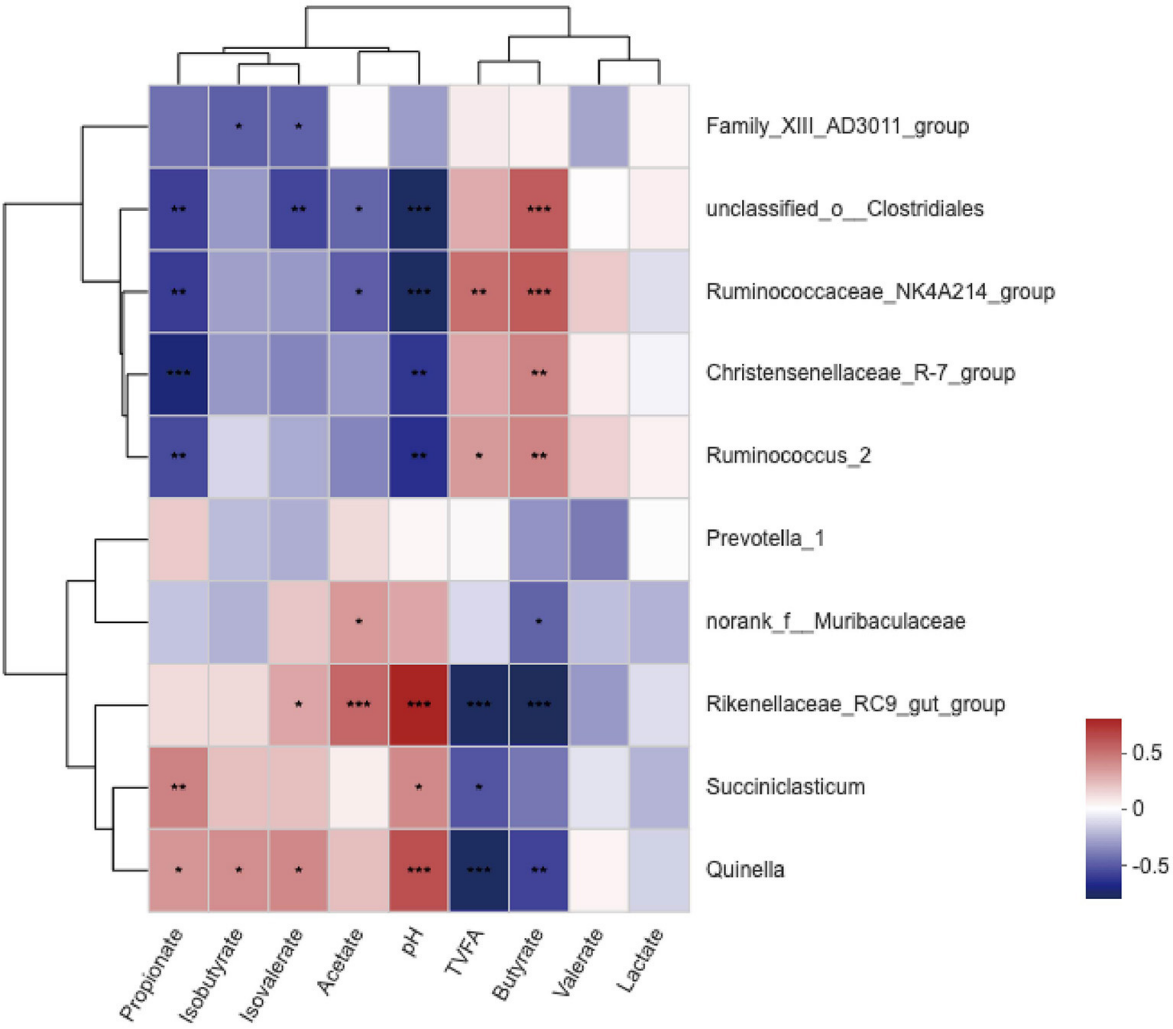
Correlation analyses between the 10 most relatively abundant bacterial genera and ruminal fermentation parameters. The blue represents a negative correlation between the abundance of the species and the VFA concentration, the red color represents a positive correlation, and the white shows that the correlation was not significant (*0.01 < *P* ≤ 0.05, **0.001 < *P* ≤ 0.01, ***P ≤ 0.001).

A correlation heat map showed that the relationships between ruminal variables and the 10 most relatively abundant bacterial genera were different (Figure 4). The ruminal pH value was positively correlated (*P* <0.05) with *Rikenellaceae_RC9__gut__group, Succiniclasticum*, and *Quinella* and negatively correlated (*P* <0.05) with *Ruminococcus_2, Ruminococcaceae_NK4A214_group, Christensenellaceae_R-7_group*, and *unclassified_o__Clostridiales*. The molar proportion of total volatile fatty acids (TVFA) showed a significant negative correlation (*P* <0.05) with *Rikenellaceae_RC9__gut__group, Succiniclasticum*, and *Quinella* and a positive correlation (*P* <0.05) with *Ruminococcaceae_NK4A214_group* and *Ruminococcus_2*. The molar proportion of butyrate was negatively correlated (*P* <0.05) with *norank_f_Muribaculaceae, Rikenellaceae_RC9__gut__group*, and *Quinella*, but was positively correlated (*P* <0.05) with *Ruminococcus_2, Ruminococcaceae_NK4A214_group, Christensenellaceae_R-7_group*, and *unclassified_o __Clostridiales*. The molar proportion of propionate demonstrated a significantly negative correlation (*P* <0.05) with *Ruminococcus_2, Ruminococcaceae_NK4A214_group, Christensenellaceae_R-7_group*, and *unclassified_o__Clostridiales* and a positive correlation (*P* <0.05) with *Succiniclasticum* and *Quinella*. The molar proportion of acetate was negatively correlated (*P* <0.05) with *Ruminococcaceae_NK4A214_group* and *unclassified_o__Clostridiales*, but was positively correlated (*P* <0.05) with *Rikenellaceae_RC9__gut__group* and *norank_f_Muribaculaceae*. The molar proportion of isobutyrate revealed a significantly positive correlation (*P* <0.05) with *Quinella* and a significantly negative correlation (*P* <0.05) with *Family_XIII__AD3011__group*. The molar proportion of isovalerate was negatively related (*P* <0.05) with *Family_XIII__AD3011__group* and *unclassified_o__Clostridiales*, but was positively related (*P* <0.05) with *Rikenellaceae_RC9__gut__group* and *Quinella*. No significant correlations (*P* > 0.05) were found between the relative abundance of genera and valerate or lactate.

### Metabolic Functions Altered in Carbohydrates of Ruminal Bacteria

The significance changed in carbohydrate metabolic pathways of the rumen bacterial microbiota in level 3 KEGG (Table 6). There were no significant differences (*P* > 0.05) in glycolysis/gluconeogenesis, amino sugar and nucleotide sugar metabolism, butanoate metabolism, galactose metabolism, propanoate metabolism, inositol phosphate metabolism, and ascorbate and aldarate metabolism among the treatments. The relative abundance of pyruvate metabolism and pentose phosphate pathway was higher in the Con treatment than the TA15, TA25, and TA35 treatments (*P* <0.05). The relative abundance of fructose and mannose metabolism, citrate cycle (TCA cycle), and glyoxylate and dicarboxylate metabolism was lower in the Con treatment than the TA15, TA25, and TA35 treatments (*P* <0.05). The relative abundance of starch and sucrose metabolism as well as pentose and glucuronate interconversions decreased with TA treatment, where it reached a significant level between the TA25, TA35, and the Con treatments. The relative abundance of C5-branched dibasic acid metabolism increased with TA treatment, where it reached a significant level between the TA35 and the Con treatments.

**Table 6:** Effects of tannic acid concentration treatments of ground corn on the altered abundances of carbohydrate metabolic pathways (%) in level 3 KEGG.

**Items**	**TA treatments**				**SEM**	***P*** **-value**	
	**0**	**0.015**	**0.025**	**0.035**		**Linear**	**Quadratic**
Glycolysis/gluconeogenesis	1.64	1.64	1.63	1.63	0.013	0.136	0.960
Amino sugar and nucleotide sugar metabolism	1.60	1.61	1.60	1.60	0.009	0.827	0.451
Pyruvate metabolism	1.61	1.57	1.57	1.57	0.008	0.000	0.056
Starch and sucrose metabolism	1.31	1.29	1.26	1.26	0.023	0.016	0.808
Pentose phosphate pathway	1.16	1.13	1.12	1.12	0.012	0.000	0.167
Glyoxylate and dicarboxylate metabolism	1.01	1.03	1.03	1.03	0.006	0.000	0.188
Fructose and mannose metabolism	0.93	0.95	0.96	0.96	0.009	0.006	0.254
Butanoate metabolism	0.94	0.94	0.9	0.96	0.011	0.044	0.700
Citrate cycle (TCA cycle)	0.90	0.93	0.94	0.94	0.011	0.000	0.094
Galactose metabolism	0.83	0.84	0.83	0.83	0.016	0.372	0.770
Propanoate metabolism	0.83	0.83	0.83	0.83	0.006	0.778	0.814
C5-branched dibasic acid metabolism	0.428	0.435	0.438	0.440	0.005	0.023	0.306
Pentose and glucuronate interconversions	0.43	0.42	0.41	0.41	0.008	0.005	0.440
Inositol phosphate metabolism	0.11	0.12	0.12	0.12	0.003	0.180	0.401
Ascorbate and aldarate metabolism	0.09	0.09	0.09	0.09	0.002	0.107	0.549

*Con, water treatment; TA, tannic acid treatment; SEM, standard error of the mean. Data represent the means of 9 replicates (n = 9)*.

### Correlations Between Bacterial Genera and Altered Carbohydrate Metabolic Pathway

**Figure 5:**
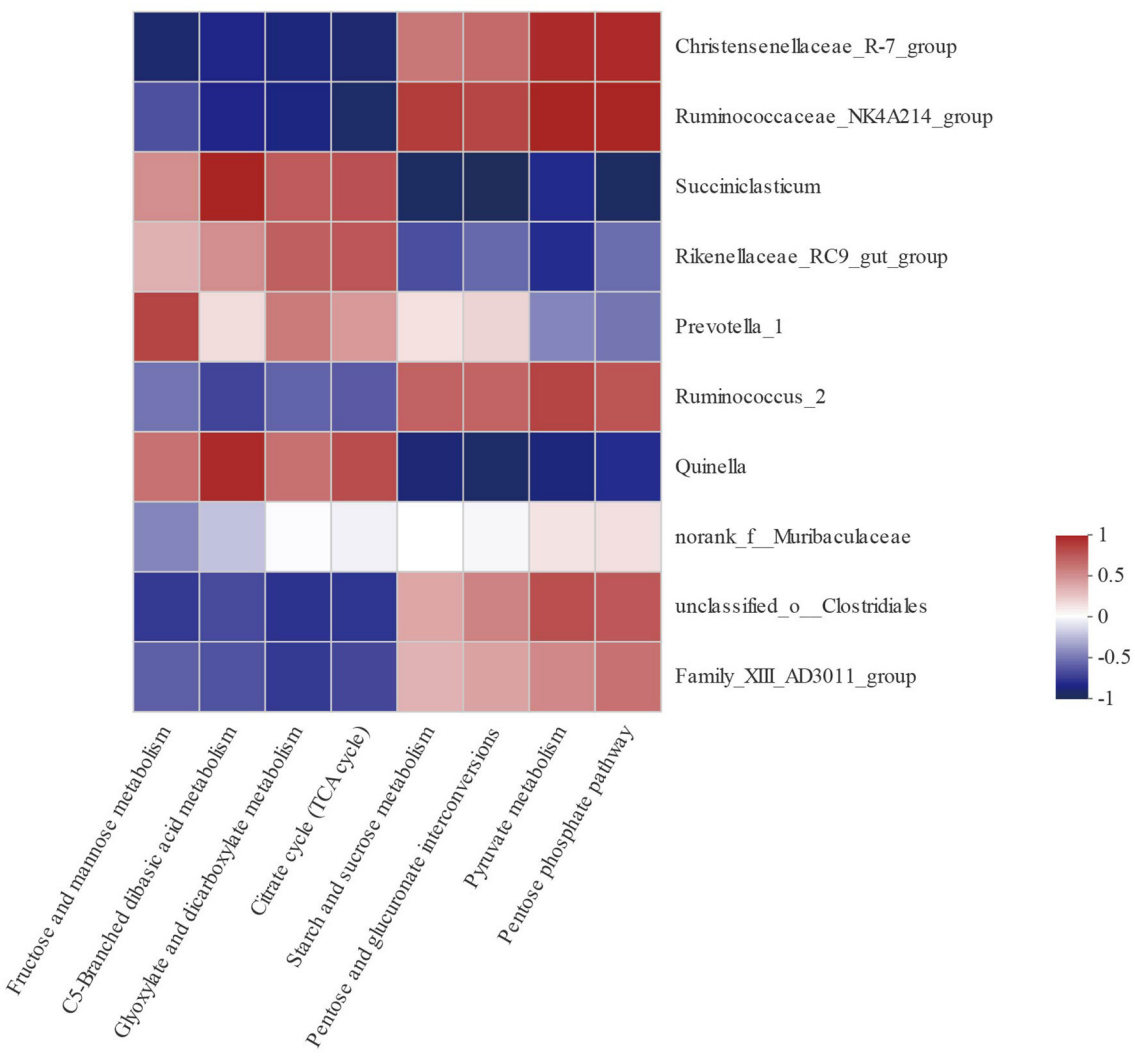
Correlation analyses between the 10 most relatively abundant bacterial genera and altered carbohydrate metabolic pathway. The blue represents a negative correlation between the abundance of the species and the metabolic pathways, the red color represents a positive correlation, and the white shows that the correlation was not significant.

In order to explore the relationships between bacterial genera and carbohydrate metabolic pathways, a Pearson correlation analysis was carried out (Figure 5). As shown in Table 7, *Christensenellaceae_R-7_group, Ruminococcaceae_NK4A214_group, Succiniclasticum, Rikenellaceae_RC9_gut_group, Prevotella_1, Ruminococcus_2, Quinella, unclassified_o_Clostridiales*, and *Family_XIII_AD3011_group* were associated with multiple metabolic pathways. For example, starch and sucrose metabolism as well as pyruvate metabolism were negatively correlated with *Succiniclasticum, Quinella*, and *Rikenellaceae_RC9_gut_group* (*P* <0.05), but positively correlated with *Christensenellaceae_R-7_group, Ruminococcaceae_NK4A214_group, Ruminococcus_2*, and *unclassified_o_Clostridiales* (*P* <0.05).

**Table 7:** Significantly positive and negative correlations between the 10 most relatively abundant bacterial genera and altered carbohydrate metabolic pathway.

**Items**	**Positive correlation**	**Negative correlation**
Pyruvate metabolism	*Christensenellaceae_R-7_group* *Ruminococcaceae_NK4A214_group* *Ruminococcus_2* *unclassified_o_Clostridiales* *Family_XIII_AD3011_group*	*Prevotella_1* *Succiniclasticum* *Quinella* *Rikenellaceae_RC9_gut_group*
Starch and sucrose metabolism	*Christensenellaceae_R-7_group* *Ruminococcaceae_NK4A214_group* *Ruminococcus_2* *unclassified_o_Clostridiales*	*Succiniclasticum* *Quinella* *Rikenellaceae_RC9_gut_group*
Pentose phosphate pathway	*Christensenellaceae_R-7_group* *Ruminococcaceae_NK4A214_group* *Ruminococcus_2* *unclassified_o_Clostridiales* *Family_XIII_AD3011_group*	*Prevotella_1* *Succiniclasticum* *Quinella* *Rikenellaceae_RC9_gut_group*
Glyoxylate and dicarboxylate metabolism	*Prevotella_1* *Succiniclasticum* *Quinella* *Rikenellaceae_RC9_gut_group*	*Christensenellaceae_R-7_group* *Ruminococcaceae_NK4A214_group* *Ruminococcus_2* *unclassified_o_Clostridiales* *Family_XIII_AD3011_group*
Fructose and mannose metabolism	*Prevotella_1* *Succiniclasticum* *Quinella*	*Christensenellaceae_R-7_group* *Ruminococcaceae_NK4A214_group* *Ruminococcus_2* *norank_f__Muribaculaceae* *unclassified_o_Clostridiales* *Family_XIII_AD3011_group*
Citrate cycle (TCA cycle)	*Prevotella_1* *Succiniclasticum* *Quinella* *Rikenellaceae_RC9_gut_group*	*Christensenellaceae_R-7_group* *Ruminococcaceae_NK4A214_group* *Ruminococcus_2* *unclassified_o_Clostridiales* *Family_XIII_AD3011_group*
C5-Branched dibasic acid metabolism	*Rikenellaceae_RC9_gut_group* *Succiniclasticum* *Quinella*	*Christensenellaceae_R-7_group* *Ruminococcaceae_NK4A214_group* *Ruminococcus_2* *unclassified_o_Clostridiales* *Family_XIII_AD3011_group*
Pentose and glucuronate interconversions	*Christensenellaceae_R-7_group* *Ruminococcaceae_NK4A214_group* *Ruminococcus_2* *unclassified_o_Clostridiales* *Family_XIII_AD3011_group*	*Succiniclasticum* *Quinella* *Rikenellaceae_RC9_gut_group*

## Discussion

In the present study, the results of ruminal fermentation showed that the TA-treated corn substrates significantly altered the ruminal fermentation profile *in vitro*. The TVFA concentration was significantly decreased by TA-treated corn accompanied by an increase in the ruminal pH value, suggesting the potential effect of tannic acid pre-treated corn in alleviating ruminal environment disorder by slowing the rate of starch degradation. Ruminal butyric subacute ruminal acidosis was induced in sheep with an intraruminal supply of ground corn, whereas lactic acidosis and propionic subacute ruminal acidosis were respectively provoked by wheat and beet pulp (35). The significant decrease in molar proportion of butyrate in TA treatments explains to some extent the potential effect of TA-treated corn in alleviating ruminal acidosis induced by high corn grain. Furthermore, the significant decrease in molar proportion of butyrate in TA treatments may be an explanation for the increasing acetate in response to TA-treated corn, because acetyl-CoA acted as substrate for producing acetate and butyrate in rumen. In addition, some studies have shown that tannins increased the proportion of acetate (36, 37) and decreased the proportion of butyrate (36). Ruminal fermentation characteristics are directly affected by the ruminal microbiota, where varied concentrations of VFA and other metabolites are dependent on the microbial population. In order to understand the relationship of TA pre-treated corn and ruminal fermentation profile, high-throughput sequencing was used to investigate the response of bacterial community to high tannic acid pre-treated corn substrates.

A number of recent studies demonstrated that high grain diet altered the community of the ruminal bacterial microbiota, decreasing the abundance of *Bacteroidetes* and increasing the abundance of *Firmicutes* under low ruminal pH (38–40). In the present study, the results of the PCoA showed that the tannic acid pre-treated corn substrates significantly altered the composition and structure of the rumen bacteria. At the phyla level, the high-grain substrates had a higher abundance of *Firmicutes* and lower *Bacteroidetes*, while the abovementioned phyla change was reversed by TA-treated corn substrates, indicating TA-treated corn substrates could stabilize the bacterial community in the rumen. Besides, we found that TA-treated corn substrates significantly decreased the relative abundance of *Patescibacteria* in respect to the Con treatment. However, little information about this phylum has been reported in the literature, but the reason for alteration in the status of this phylum remains unclear.

Significantly, shifts in the genera S*ucciniclasticum, Ruminococcus_2, Ruminococcaceae_NK4A214_group, Quinella*, and *Rikenellaceae_RC9_gut_group* were found in the present study. *Succiniclasticum* only ferments succinate to propionate but not other carbohydrates; amino acids; or mono-, di-, and other tricarboxylic acids (41). As the primary succinate-utilizing bacteria, *Succiniclasticum* accounted for 5.34% of total bacterial community in the Con group and decreased significantly compared with the TA25 and TA35 groups. Therefore, the increase of *Succiniclasticum* needs more pyruvate to metabolize succinate for producing propionate. The above observation fits with the results that there is more molar proportion of propionate in TA treatment corn substrate group. Meanwhile, a positive correlation between the relative abundance of *Succiniclasticum* and the molar proportion of propionate was found in the current study. As for acetate-producing bacteria, we observed that there was a decrease in the relative abundance of *Ruminococcus_2* and *Ruminococcaceae_NK4A214_group* in TA treatments. It is somewhat surprising that the molar proportion of acetate was still increased in the TA groups. The high abundance of *Rikenellaceae_RC9_gut_group* may be the possible explanation for this finding. Collins et al. (42) and Su et al. (43) reported that acetate is a major product of glucose fermentation in the family *Rikenellaceae*. These explanations became plausible as the significantly positive correlation between the relative abundance of *Rikenellaceae_RC9_gut_group* and the molar proportion of acetate was observed. The decreased abundance of *Christensenellaceae_R-7_group* in TA-treated corn groups demonstrated that the TA-treated corn inhibits growth of this genera. Further research should be undertaken to investigate why TA treatment reduced the relative abundance of this genera. Lactic acid is produced as the major fermentation product by *Quinella*, when sugars do not limit growth. When sugars are limited and growth is slow, acetate and propionate are the major fermentation products (44). Lactate can also be produced by Clostridiales, Ruminococcaceae, Lachnospiraceae, and *Butyrivibrio* in the rumen (45). In the current study, the significant changes in the relative abundance of *Quinella, Ruminococcaceae_NK4A214_group*, and *unclassified_o__Clostridiales* should have caused a shift in lactate concentration. Surprisingly, lactate concentration was not found different among the groups. Meanwhile, no correlation was found between bacterial genera and lactate. The results above might be explained by the fact that as the intermediate product of carbohydrate metabolism, lactate can be metabolized by lots of bacterial genera for their growth. This combination of competitive process may lead to the disappearing of change in lactate concentration. Therefore, causes for this phenomenon are not well-understood and need to be further studied to get a more precise explanation. Among various relevant studies, high-grain diet supplementation increased, decreased, or had no effect on the amount of rumen *Prevotella* (46–49). Our result showed that the relative abundance of *Prevotella_1* was not affected by TA treatments compared with the Con group. The members of *Prevote*lla are characterized by various metabolic potentials and possess multiple degradation activities, including those of starch, fiber, hemicellulose, protein, and sugar (50, 51). These results suggested that *Prevotella* spp. may be more tasteful than expected due to its multiple activities leading to adaptive responses to the rumen substrate changes, and these changes did not exhibit specific patterns.

Our analysis using PICRUSt found that lots of carbohydrate metabolic pathways in level 3 KEGG were differential. The metabolites of starch and sucrose metabolism, pentose phosphate pathway, glyoxylate and dicarboxylate metabolism, as well as fructose and mannose metabolism (carbohydrate metabolism) can enter glycolysis/gluconeogenesis and pyruvate metabolism pathways (52–55). Importantly, TA-treated corn reduced pyruvate metabolism as well as starch and sucrose metabolism compared with the control group. Meanwhile, pyruvate metabolism as well as starch and sucrose metabolism were negatively correlated with *Christensenellaceae_R-7_group, Ruminococcaceae_NK4A214_group, Ruminococcus_2*, and *unclassified_o_Clostridiales*. These results indicated that TA- treated corn could weaken starch and pyruvate metabolism of microbiota. In rumen, pyruvate produced by starch can be utilized to produce acetate, butyrate (acetate and butyrate pathway), and propionate (succinate pathway and acrylate pathway). The decrease in pyruvate metabolism leads to the reduction of VFA and increase ruminal pH produced by ruminal microbiota in TA treatments, which may be attributed to the slow degradation starch. Furthermore, the results presented earlier seem to indicate that tannic acid pre-treated corn has a potential to improve ruminal environment by slowing the rate of starch degradation.

## Conclusion

The present study investigated the effects of TA-treated corn on the rumen fermentation characteristics and microbial composition *in vitro*. In summary, TA-treated corn discriminatively altered the ruminal fermentation, microbial composition, and diversity and modulated the carbohydrate metabolic pathways of microbial metabolism. These alterations may help us for a better understanding of the mechanism of TA-treated corn to regulate rumen environment disorder induced by high-grain substrate.

## Data Availability Statement

The datasets presented in this study can be found in online repositories. The names of the repository/repositories and accession number can be found at: https://www.ncbi.nlm.nih.gov/, PRJNA768092.

## Author Contributions

FZ and HW designed the research. FZ, XZ, and YQ conducted the research. FZ, YZ, HS, and HZ analyzed the data. FZ wrote the manuscript. ME revised the language. All authors contributed to the article and approved the submitted version.

## Funding

The authors would like to acknowledge the funding received to conduct this study from the project funded by the National Natural Science Foundation of China (NSFC No. 31872988 and No. 31572429), the Postgraduate Research & Practice Innovation Program of Jiangsu Province (KYCX18_2375), and the Priority Academic Program Development of Jiangsu Higher Education Institutions (PAPD).

## Conflict of Interest

The authors declare that the research was conducted in the absence of any commercial or financial relationships that could be construed as a potential conflict of interest.

## Publisher's Note

All claims expressed in this article are solely those of the authors and do not necessarily represent those of their affiliated organizations, or those of the publisher, the editors and the reviewers. Any product that may be evaluated in this article, or claim that may be made by its manufacturer, is not guaranteed or endorsed by the publisher.
